# Expression of Concern: microRNA-98 inhibits the proliferation, invasion, migration and promotes apoptosis of breast cancer cells by binding to HMGA2

**DOI:** 10.1042/BSR-20180571_EOC

**Published:** 2020-09-28

**Keywords:** Breast cancer, High mobility group AT-hook 2, Invasion, microRNA-98, Migration, Proliferation

The Editorial Office has been made aware of potential issues surrounding the scientific validity of this paper, hence has issued an expression of concern to notify readers whilst the Editorial Office investigates.

